# Biostimulant Effects of Plant-Growth-Promoting Bacteria (PGPB) Consortia in *Zea mays*, *Brassica oleracea,* and *Solanum lycopersicum*

**DOI:** 10.3390/plants15193028

**Published:** 2026-10-03

**Authors:** Rosa Evelia Fernández-Coronado, Elías Octavio Gómez-Montes, Aslinn Fernanda Martínez-Pérez, Vania Jhoana Muñoz-Serralde, Paola Monserrat Martínez-Avelino, Fabián Robles-Martínez, Ana Belem Piña-Guzmán

**Affiliations:** 1 Unidad Profesional Interdisciplinaria de Biotecnología (UPIBI), Instituto Politécnico Nacional (IPN), Av. Acueducto S/N, Barrio la Laguna Ticomán, Delegación Gustavo A. Madero, Ciudad de México 07340, Mexico; rosaefc@yahoo.com.mx (R.E.F.-C.); aslinmartinez2@gmail.com (A.F.M.-P.); vaniamunoz1804@gmail.com (V.J.M.-S.); paola.martinezavelino@gmail.com (P.M.M.-A.); 2 Escuela de Ingeniería y Ciencias, Instituto Tecnológico y de Estudios Superiores de Monterrey (ITESM-CEM), Carretera Lago de Guadalupe Km 3.5, Atizapán de Zaragoza 52926, Mexico; octavio.gomez@tec.mx

**Keywords:** crops improvement, PGPB, *Azospirillum brasilense*, *Pseudomonas putida*, *Bacillus* sp., *Mycobacterium* sp.

## Abstract

The overuse of chemical fertilizers highlights the need for sustainable alternatives, such as plant-growth-promoting bacteria (PGPB). However, bacterial inoculant performance may vary with plant species and growth conditions. This study evaluated the effects of microbial consortia combining *Azospirillum brasilense*, *Pseudomonas putida*, *Bacillus* sp., and *Mycobacterium* sp. on blue maize, broccoli, and tomato grown in commercial substrate and greenhouse soil. Consortium effects on early plant growth varied according to bacterial combination, plant species and substrate conditions. *Pseudomonas putida* + *Bacillus* sp. increased blue maize main root length by 28.45% in greenhouse soil and stem length by 44.70% in commercial substrate, while increasing tomato shoot dry weight by over 500% in greenhouse soil. *Azospirillum brasilense* + *Bacillus* sp. increased shoot dry weight by 154.06% in broccoli and 274.87% in tomato, both in commercial substrate. Results obtained with consortia containing *Mycobacterium* sp. provide exploratory evidence strictly under laboratory conditions. Due to the potential health risk associated with opportunistic and pathogenic species within this genus, these strains cannot be considered for agricultural applications or biofertilizer development, serving exclusively as a model for studies. Overall, these findings emphasize that PGPB consortium performance was context-dependent, emphasizing the importance of plant species, substrate conditions, and microbial composition when evaluating bacterial inoculants.

## 1. Introduction

Agriculture is one of the most important economic activities worldwide. In Mexico, staple and horticultural crops such as maize (*Zea mays*), broccoli (*Brassica oleracea*), and tomato (*Solanum lycopersicum*) play an important role in both national consumption and international markets. Per capita consumption of these crops was reported at 164 kg of white maize, 1.6 kg of broccoli, and 14 kg of tomatoes in 2024, while Mexico is a leading global producer, ranking seventh in white maize production, fourth in broccoli production and seventh in tomato production [1]. Due to high demand, the use of fertilizers remains widespread; in 2022, approximately 65.5% of large-scale open-field agricultural producers in Mexico applied synthetic fertilizers [2]. However, the intensive use of chemical fertilizers has been associated with adverse environmental consequences, including greenhouse gas emissions, soil and water contamination, and the accumulation of chemical residues in food crops [3].

In response to these challenges, sustainable agricultural alternatives such as plant-growth-promoting bacteria (PGPB) have gained increased attention. PGPB are beneficial microorganisms that reside in the rhizosphere and plant roots. They can modulate plant development through direct and indirect mechanisms, including phytohormone production, nutrient solubilization, biological nitrogen fixation, and the induction of systemic resistance [4].

Plants coexist with multiple microbial consortia in their environment. Although many microorganisms remain neutral or can even be pathogenic, plants have the evolutionary ability to recruit beneficial microorganisms. This recruitment process occurs through different mechanisms, including vertical transmission of endophytic cells from one generation to the next and actively aiding bacterial colonization through the secretion of root exudates [5]. Among the vast diversity of rhizosphere microorganisms, several genera and species have been extensively studied worldwide for their remarkable plant-growth-promoting capabilities. The individual use of PGPMs has been extensively studied, though their performance varies among plant species and environmental conditions. Reported effects include increases in root length, foliage development, and grain yield [6]. In addition, PGPMs are known to improve plant tolerance to biotic and abiotic stresses [7], while contributing to soil health through mechanisms such as biological nitrogen fixation, phosphate solubilization, potassium solubilization, and phytohormone production [8]. Some of the most studied genera and strains are described below.

*Bacillus*, for instance, is one of the most prominent genera in agricultural research, widely recognized for its excretion of antibiotics, siderophore-mediated iron regulation, production of lytic enzymes, and ISR (induced systemic resistance) [9]. Representative strains, such as *Bacillus* sp. CDBB B-551, naturally exemplify versatile traits of biocontrol and biofertilization. The genus *Mycobacterium* has also attracted considerable attention. Specific strains, including *Mycobacterium* sp. CDBB B-77, have been demonstrated to synthesize crucial phytohormones such as gibberellins [10] and indole-3-acetic acid (IAA) primarily through the L-tryptophan metabolic pathway [11].

In terms of nitrogen fixation and root architecture modulation, *Azospirillum brasilense* stands out as one of the most rigorously researched PGPB. Strains such as CDBB B-1887 are highly valued for their robust IAA production, a phytohormone essential for the longitudinal growth of stems and roots, which also contributes to increased plant dry weight and enhanced drought resistance [12]. Conversely, *Pseudomonas putida* (e.g., strain CDBB B-1299) is frequently highlighted in the literature for its dual functionality; it not only promotes the formation of IAA but also plays a critical role in mitigating abiotic stresses such as drought, while triggering the induction of systemic plant resistance [13].

The quality of soil and plants is significantly influenced by these soil microorganisms [14]. Phylogenetically diverse microbial communities help modulate phytohormone levels in the rhizosphere, causing various positive effects such as promoting plant growth, contributing to the host’s drought tolerance, and aiding nitrogen fixation in the soil [15].

Despite the well-documented benefits of individual PGPB strains, the behavior of defined multi-strain consortia under varying soil conditions is not yet fully understood. Previous studies have often focused on natural microbial consortia found in soils [16] or on commercial substrates under controlled conditions [17], highlighting the need for further evaluation of defined multi-strain PGPB consortia across diverse soils or substrates and plant species under controlled greenhouse conditions [18]. In this study, we hypothesized that the application of multi-strain PGPB consortia would result in strain-dependent improvements in vegetative growth and biomass in selected agricultural crops, and that these responses would vary depending on the soil or substrate and the plant species. Therefore, this study aims to assess the impact of a consortium composed of four diverse PGPB (*Azospirillum brasilense*, *Pseudomonas putida*, *Bacillus* sp., and *Mycobacterium* sp.) on the longitudinal growth of main roots, stems, and dry weight of the agriculturally important plants *Zea mays*, *Brassica oleracea* var. italica, and *Solanum lycopersicum*, comparing two contrasting growth media: a commercial substrate (perlite–peat moss mixture) and greenhouse soil, which is representative of agricultural production systems.

## 2. Results

### 2.1. Abbreviations and Nomenclature

The following nomenclature was used to name the consortia: *Pseudomonas putida* with *Bacillus* sp. (Pp + Bsp), *Pseudomonas putida* with *Azospirillum brasilense* (Pp + Azo), *Azospirillum brasilense* with *Bacillus* sp. (Azo + Bsp), *Mycobacterium* sp. with *Azospirillum brasilense* (My + Azo), *Mycobacterium* sp. with *Pseudomonas putida* (My + Pp), *Mycobacterium* sp. with *Bacillus* sp. (My + Bsp), and *Mycobacterium* sp. with *Pseudomonas putida*, *Azospirillum brasilense* and *Bacillus* sp. (My + Pp + Azo + Bsp). The two substrate types are commercial substrate (CS) and greenhouse soil (GS).

### 2.2. Bacterial Kinetics

**Figure 1:**
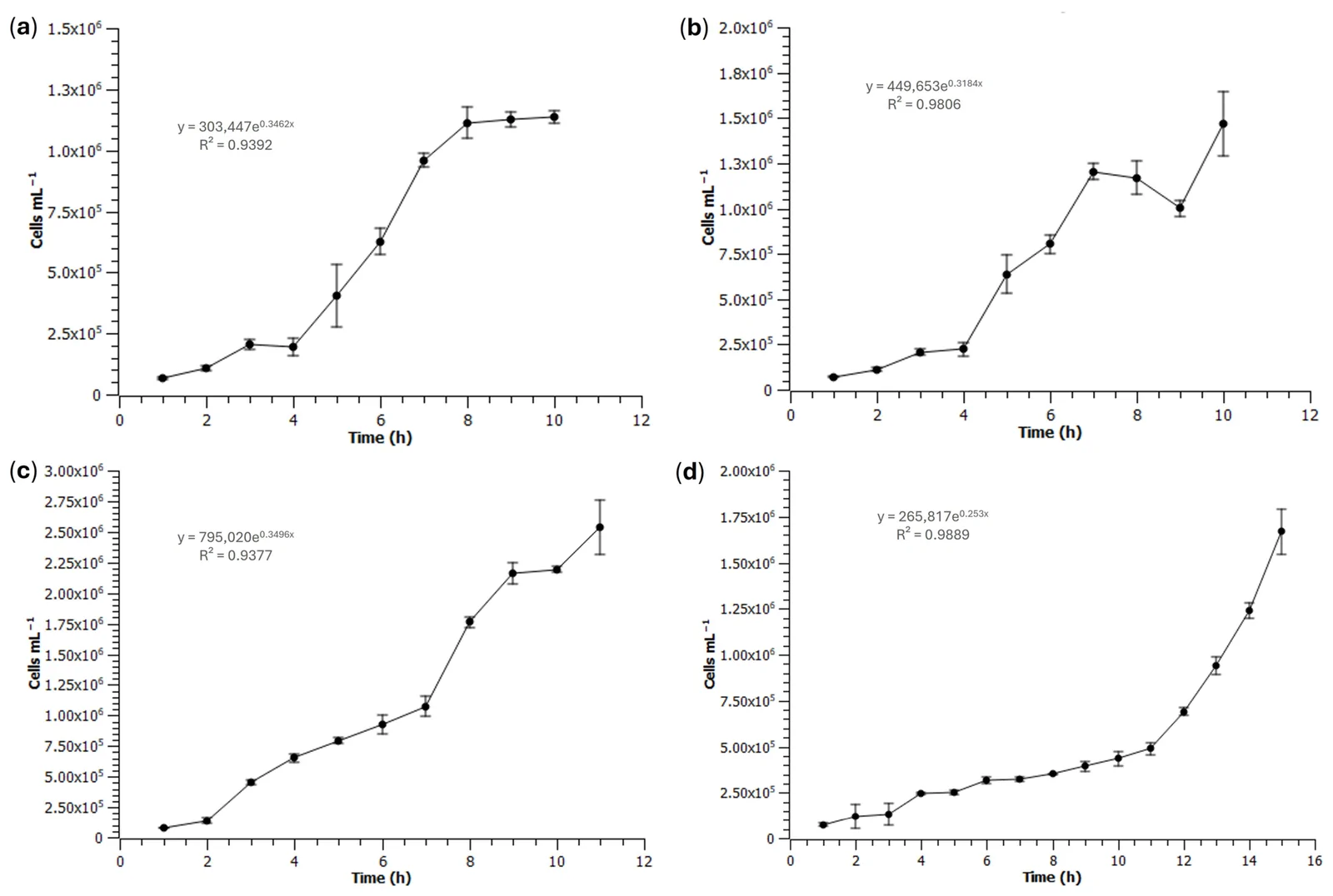
Growth kinetics of (**a**) *Azospirillum brasilense*, (**b**) *Bacillus* sp., (**c**) *Pseudomonas putida*, and (**d**) *Mycobacterium* sp. expressed as cell concentration (cells mL^−1^) over time. Mean ± standard deviation of the three independent samples. The equations were calculated using data points from the exponential growth phase.

The bacterial strains displayed distinct growth patterns during cultivation (Figure 1). Differences in incubation times required to reach the target cell density (1 × 10^6^ cells mL^−1^), as well as specific kinetic parameters for each strain, are summarized in Table 1.

### 2.3. Effect of Microbial Consortia on Longitudinal Main Root Growth

**Figure 2:**
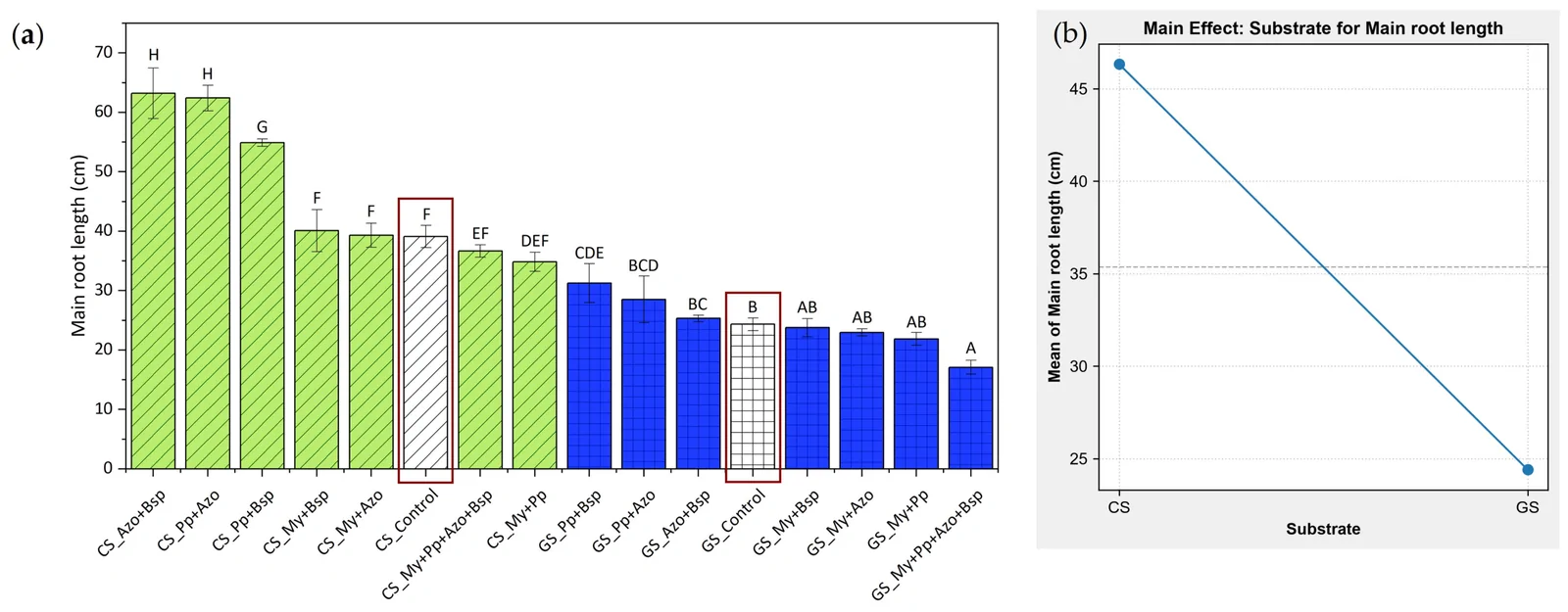
Effect of microbial consortia on main root length of blue maize. (**a**) Main root length of plants grown in commercial substrate (CS; green bars with diagonal stripes) and greenhouse soil (GS; blue bars with grid pattern). White bars outlined in red indicate the corresponding uninoculated controls (CS_Control and GS_Control). Data are presented as mean ± standard deviation (*n* = 3 replicates, with five plants per replicate). Means sharing at least one letter are not significantly different according to Tukey’s multiple comparison test (*p* < 0.05). (**b**) Main-effects plot showing the overall effect of substrate type on main root length.

Longitudinal growth of the main root in blue maize was significantly greater (*p* < 0.05) than the control in greenhouse soil by Pp + Bsp, achieving an increase of 28.45%. Meanwhile, in the commercial substrate, which exerted the greatest overall influence (Figure 2b), increases reached 40.41% for Pp + Bsp, 59.61% for Pp + Azo and 61.68% for Azo + Bsp (Figure 2a).

**Figure 3:**
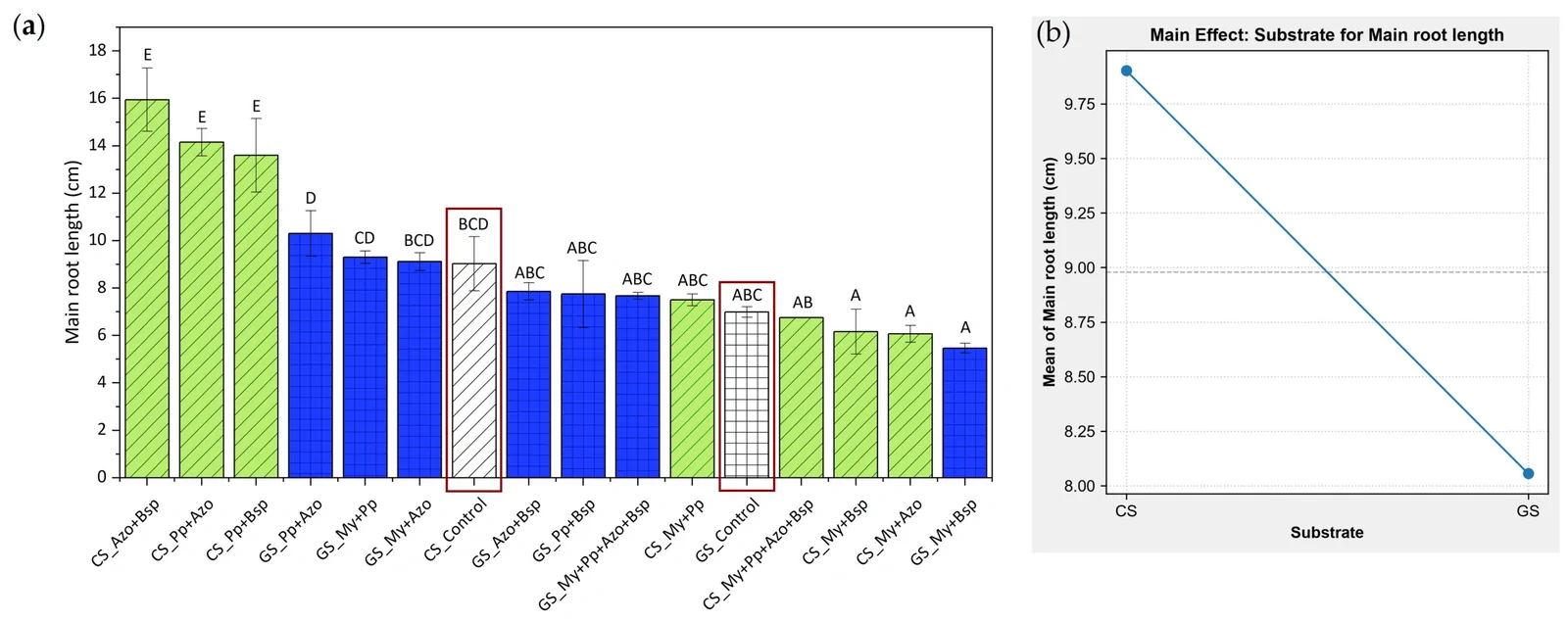
Effect of microbial consortia on main root length of broccoli. (**a**) Main root length of plants grown in commercial substrate (CS; green bars with diagonal stripes) and greenhouse soil (GS; blue bars with grid pattern). White bars outlined in red indicate the corresponding uninoculated controls (CS_Control and GS_Control). Data are presented as mean ± standard deviation (*n* = 3 replicates, with five plants per replicate). Means sharing at least one letter are not significantly different according to Tukey’s multiple comparison test (*p* < 0.05). (**b**) Main-effects plot showing the overall effect of substrate type on main root length.

For broccoli, significantly greater (*p* < 0.05) primary root lengths than the control were observed for the Pp + Bsp, Pp + Azo, and Azo + Bsp consortia in commercial substrate (50.58%, 56.70%, and 76.56%, respectively), whereas in greenhouse soil, the highest increases were recorded for the Pp + Azo, My + Pp and My + Azo consortia (47.35%, 33.04% and 30.42%, respectively) (Figure 3a). These results indicate that substrate type influenced the effectiveness of the microbial consortia, with the commercial substrate generally promoting greater increases in primary root length than greenhouse soil (Figure 3b).

**Figure 4:**
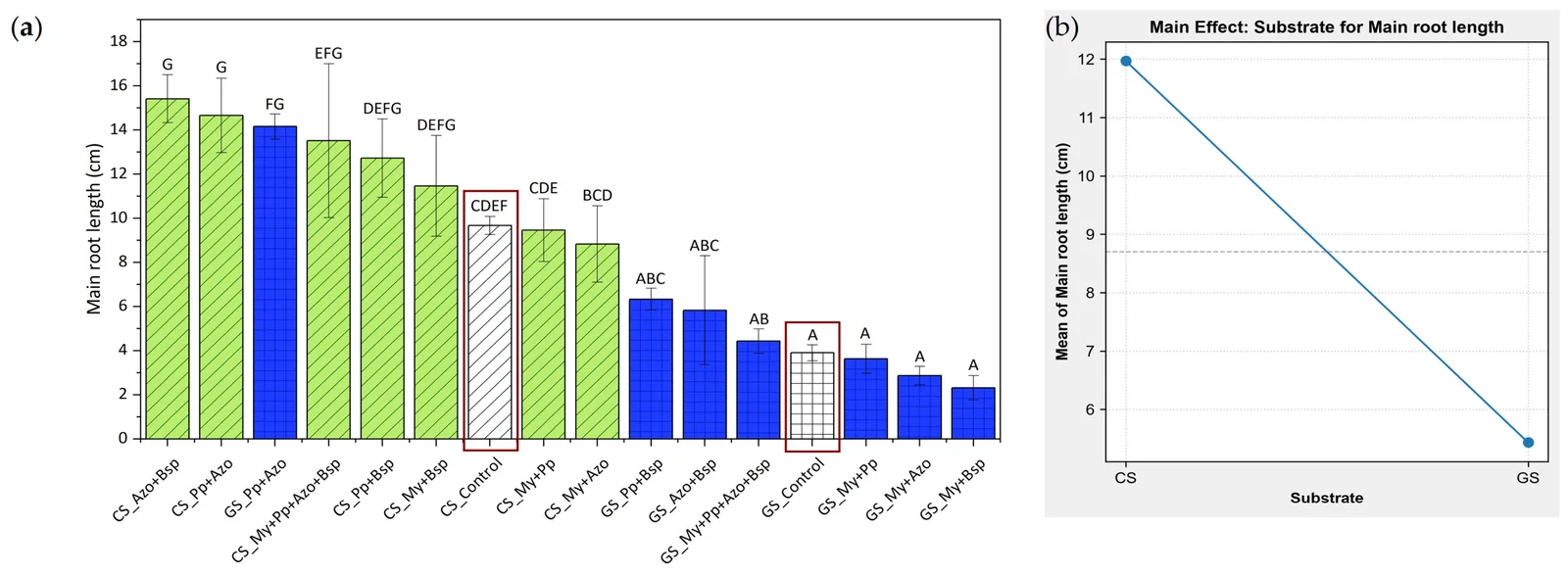
Effect of microbial consortia on main root length of tomato. (**a**) Main root length of plants grown in commercial substrate (CS; green bars with diagonal stripes) and greenhouse soil (GS; blue bars with grid pattern). White bars outlined in red indicate the corresponding uninoculated controls (CS_Control and GS_Control). Data are presented as mean ± standard deviation (*n* = 3 replicates, with five plants per replicate). Means sharing at least one letter are not significantly different according to Tukey’s multiple comparison test (*p* < 0.05). (**b**) Main-effects plot showing the overall effect of substrate type on main root length.

In tomato, the consortia that showed significantly greater (*p* < 0.05) effect versus the control in the commercial substrate in main root length were Azo + Bsp and Pp + Azo (59.38% and 51.60%, respectively). Under greenhouse soil conditions, the highest main root growth was observed in the Pp + Azo consortium (262.90%) (Figure 4a). The commercial substrate exerted the most influence (Figure 4b).

### 2.4. Effect of Microbial Consortia on Stem Plant Growth

**Figure 5:**
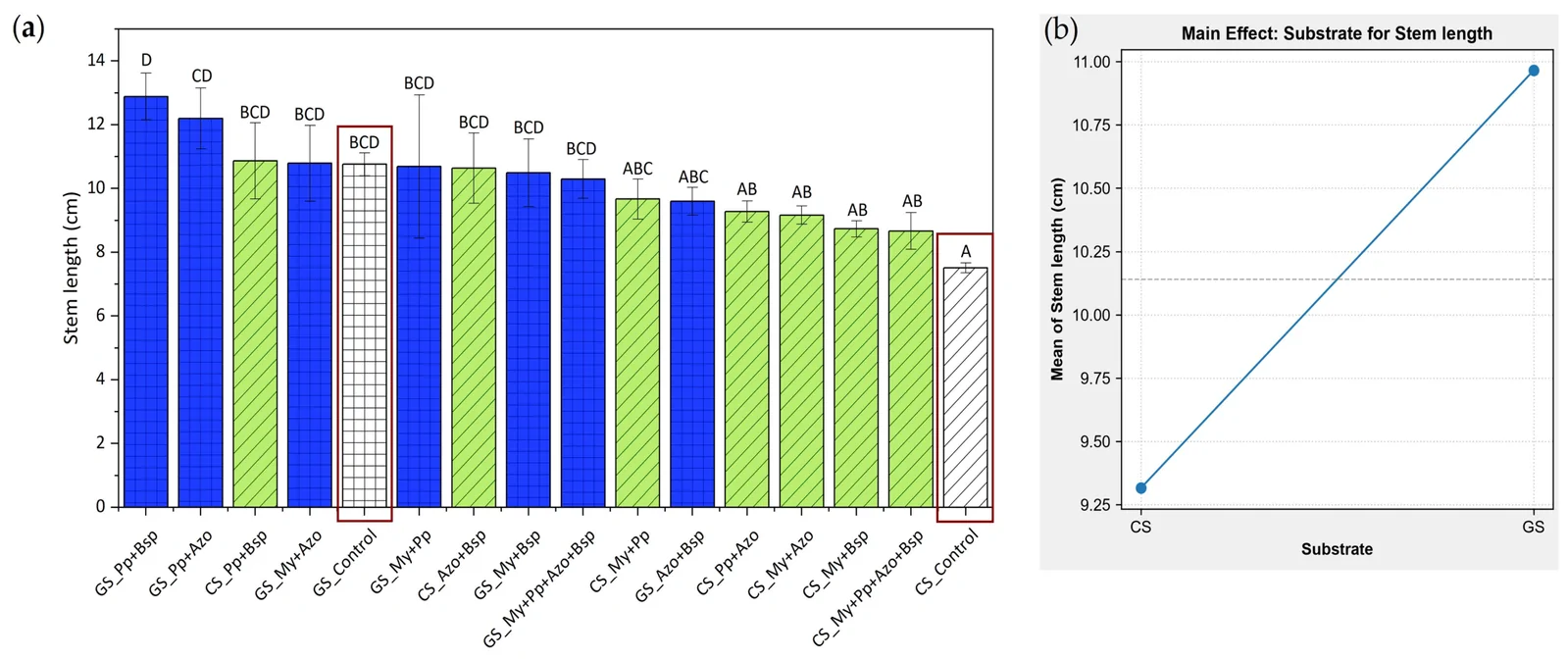
Effect of microbial consortia on stem length of blue maize. (**a**) Stem length of plants grown in commercial substrate (CS; green bars with diagonal stripes) and greenhouse soil (GS; blue bars with grid pattern). White bars outlined in red indicate the corresponding uninoculated controls (CS_Control and GS_Control). Data are presented as mean ± standard deviation (*n* = 3 replicates, with five plants per replicate). Means sharing at least one letter are not significantly different according to Tukey’s multiple comparison test (*p* < 0.05). (**b**) Main-effects plot showing the overall effect of substrate type on stem length.

In blue maize, a significantly higher increase (*p* < 0.05) in stem length compared with the control in the commercial substrate was observed for the Pp + Bsp consortium, enhancing stem growth by 44.70%, followed by Azo + Bsp with an increase of 41.59% (Figure 5a). This time the greenhouse soil was the substrate that exerted the most influence (Figure 5b).

**Figure 6:**
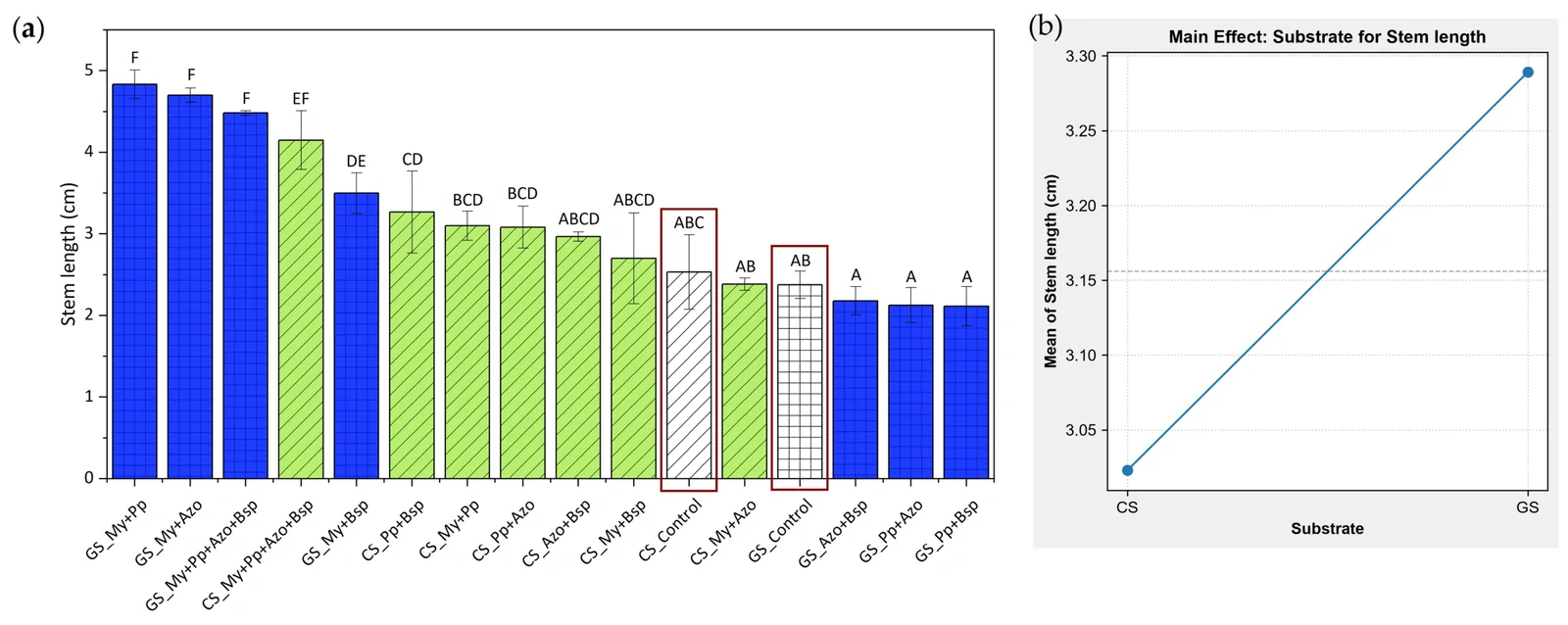
Effect of microbial consortia on stem length of broccoli. (**a**) Stem length of plants grown in commercial substrate (CS; green bars with diagonal stripes) and greenhouse soil (GS; blue bars with grid pattern). White bars outlined in red indicate the corresponding uninoculated controls (CS_Control and GS_Control). Data are presented as mean ± standard deviation (*n* = 3 replicates, with five plants per replicate). Means sharing at least one letter are not significantly different according to Tukey’s multiple comparison test (*p* < 0.05). (**b**) Main-effects plot showing the overall effect of substrate type on stem length.

In broccoli, the greatest response was obtained with the My + Pp + Azo + Bsp consortium, which increased stem growth by 63.81%, followed by Pp + Bsp with an increase of 28.94% in commercial substrate. Under greenhouse soil conditions, consortia containing *Mycobacterium* sp. showed the greatest effects, with My + Azo, My + Pp, My + Bsp and My + Pp + Azo + Bsp increasing stem growth by 97.80%, 103.41%, 47.29%, and 88.68%, respectively (Figure 6a). The greenhouse soil was the soil that exerted the most influence (Figure 6b).

**Figure 7:**
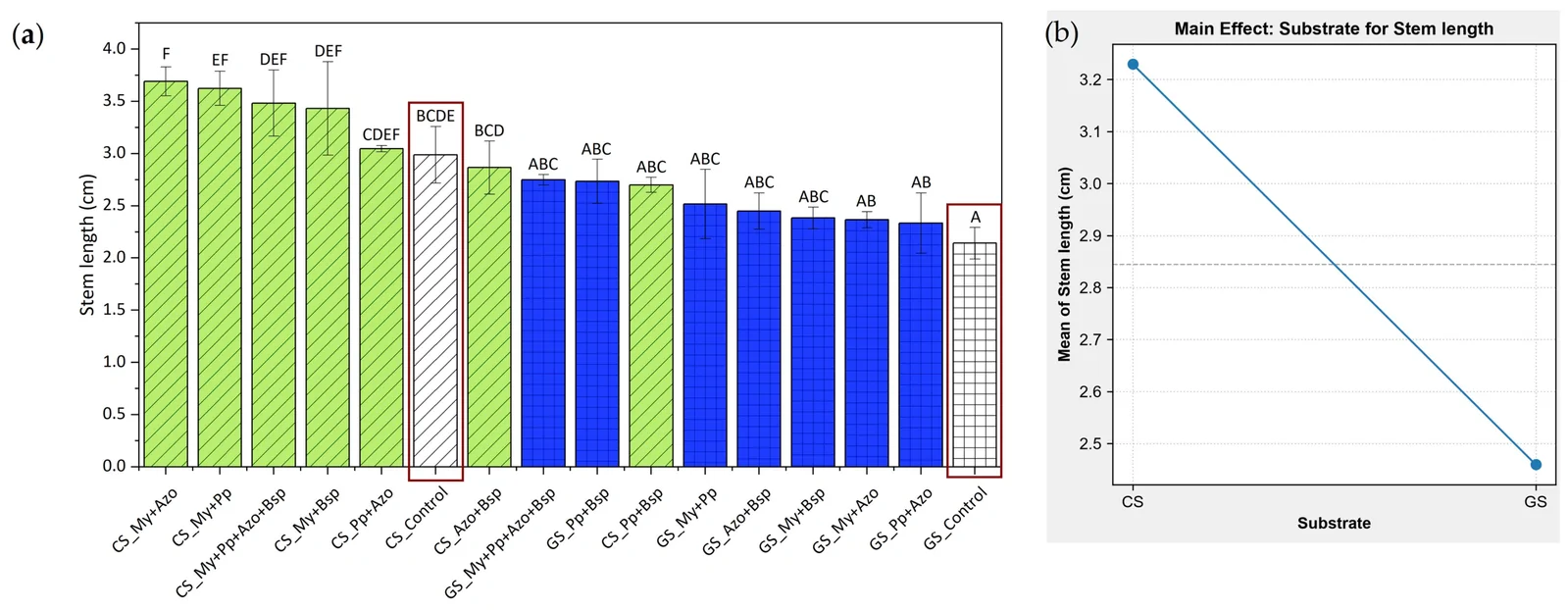
Effect of microbial consortia on stem length of tomato. (**a**) Stem length of plants grown in commercial substrate (CS; green bars with diagonal stripes) and greenhouse soil (GS; blue bars with grid pattern). White bars outlined in red indicate the corresponding uninoculated controls (CS_Control and GS_Control). Data are presented as mean ± standard deviation (*n* = 3 replicates, with five plants per replicate). Means sharing at least one letter are not significantly different according to Tukey’s multiple comparison test (*p* < 0.05). (**b**) Main-effects plot showing the overall effect of substrate type on stem length.

In tomato, consortia containing *Mycobacterium* sp. showed the greatest stem growth in the commercial substrate significantly (*p* < 0.05), particularly in My + Azo with increases of 23.57%. The consortia that contained *Mycobacterium* sp. had the greatest influence on stem growth of tomato (Figure 7a), and the commercial substrate was the medium that exerted the most influence (Figure 7b).

### 2.5. Effect of Microbial Consortia on Biomass (Dry Weight)

**Figure 8:**
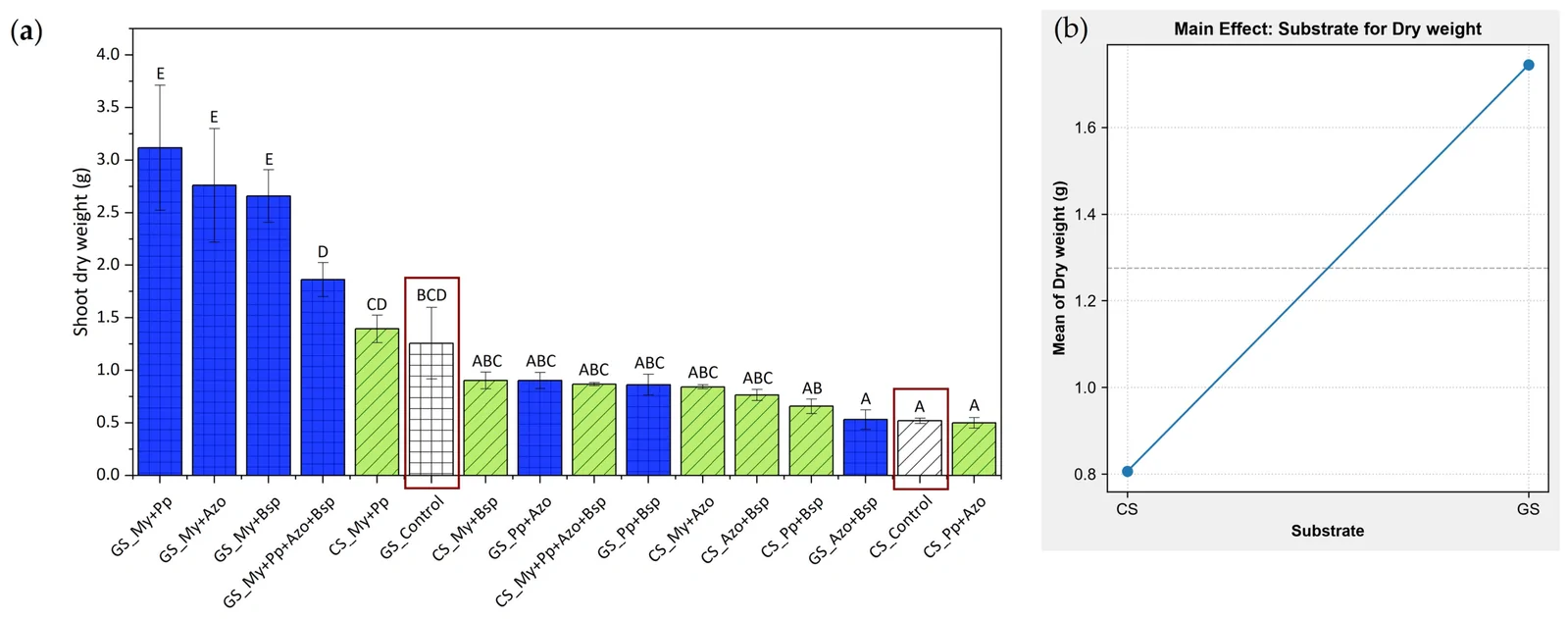
Effect of microbial consortia on biomass (dry weight) of the aerial parts of blue maize. (**a**) Biomass (dry weight) of aerial parts of plants grown in commercial substrate (CS; green bars with diagonal stripes) and greenhouse soil (GS; blue bars with grid pattern). White bars outlined in red indicate the corresponding uninoculated controls (CS_Control and GS_Control). Data are presented as mean ± standard deviation (*n* = 3 replicates, with five plants per replicate). Means sharing at least one letter are not significantly different according to Tukey’s multiple comparison test (*p* < 0.05). (**b**) Main-effects plot showing the overall effect of substrate type on biomass (dry weight) of the aerial parts.

For the dry weight parameter, blue maize plants inoculated with the Pp + Bsp consortium showed a 26.54% increase compared to the control in commercial substrate. In greenhouse soil the consortia containing *Mycobacterium* sp. consistently produced significantly higher (*p* < 0.05) dry-weight values than the control. The greatest increases were observed in the My + Pp consortium (147.85%), followed by My + Azo (119.50%) and My + Bsp (111.37%) (Figure 8a). The consortia that contained *Mycobacterium* sp. had the greatest influence on dry weight of blue maize in greenhouse soil, which was also the substrate that exerted the most influence (Figure 8b).

**Figure 9:**
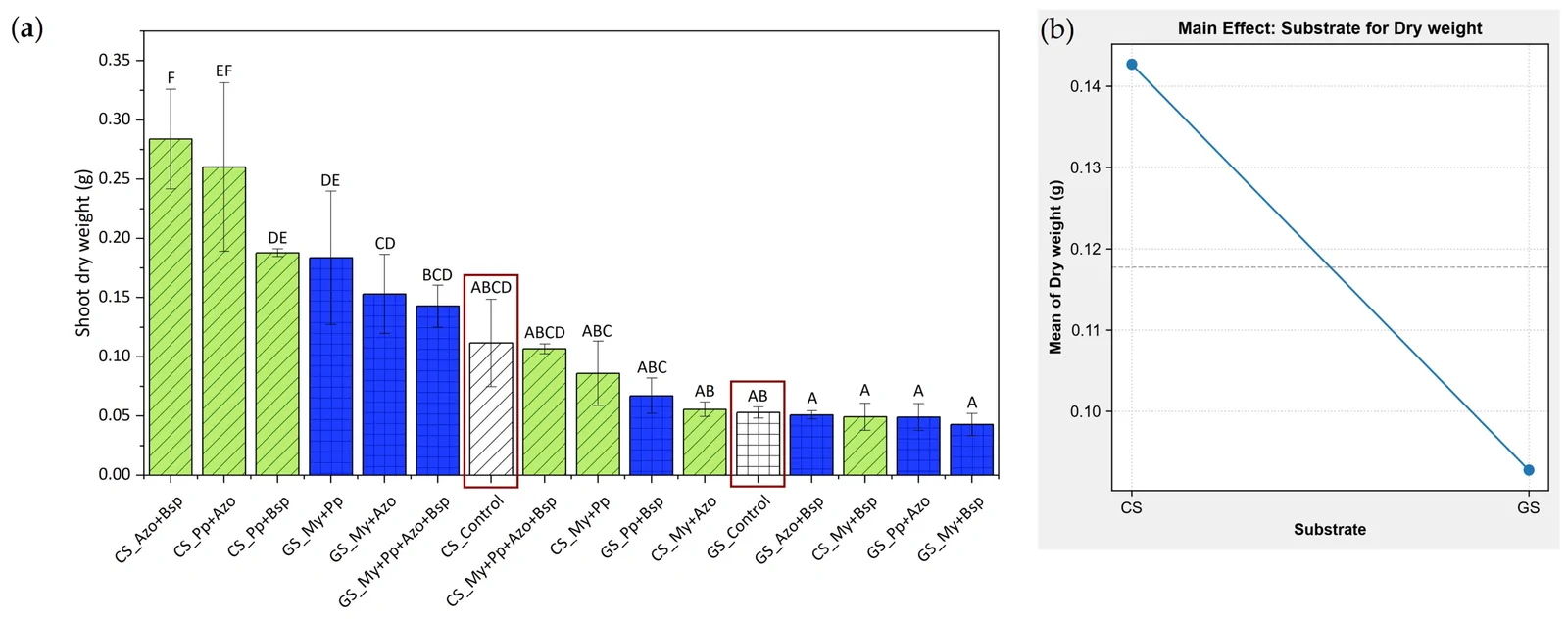
Effect of microbial consortia on biomass (dry weight) of aerial parts of broccoli. (**a**) Biomass (dry weight) of aerial parts of plants grown in commercial substrate (CS; green bars with diagonal stripes) and greenhouse soil (GS; blue bars with grid pattern). White bars outlined in red indicate the corresponding uninoculated controls (CS_Control and GS_Control). Data are presented as mean ± standard deviation (*n* = 3 replicates, with five plants per replicate). Means sharing at least one letter are not significantly different according to Tukey’s multiple comparison test (*p* < 0.05). (**b**) Main-effects plot showing the overall effect of substrate type on biomass (dry weight) of the aerial parts.

In broccoli, significant increases (*p* < 0.05) in dry weight were observed in the commercial substrate following inoculation with the Pp + Azo and Azo + Bsp consortia, resulting in biomass increases of 132.95% and 154.06%, respectively, compared with the control. In greenhouse soil, the most effective treatments were My + Azo and My + Pp, which increased dry weight by 188.92% and 246.54%, respectively (Figure 9a). The commercial substrate was the soil that exerted the most influence (Figure 9b).

**Figure 10:**
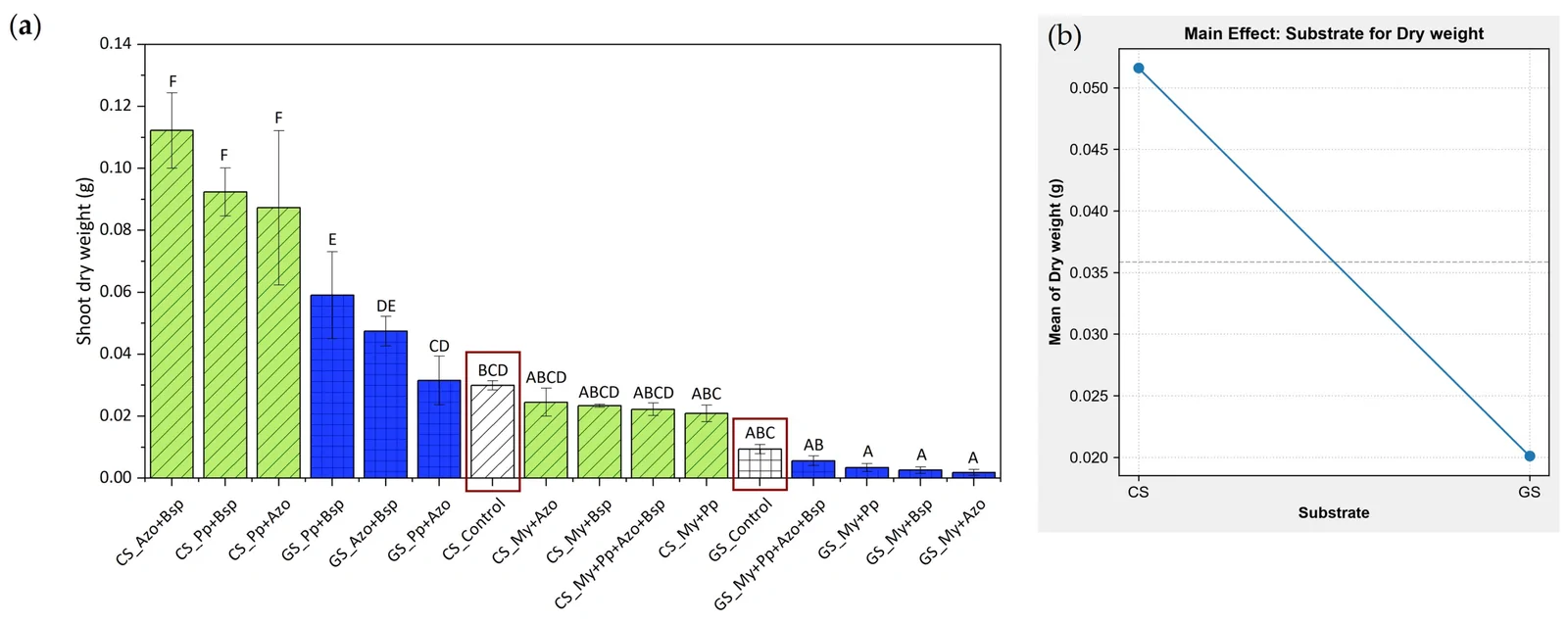
Effect of microbial consortia on biomass (dry weight) of aerial parts of tomato. (**a**) Biomass (dry weight) of aerial parts of plants grown in commercial substrate (CS; green bars with diagonal stripes) and greenhouse soil (GS; blue bars with grid pattern). White bars outlined in red indicate the corresponding uninoculated controls (CS_Control and GS_Control). Data are presented as mean ± standard deviation (*n* = 3 replicates, with five plants per replicate). Means sharing at least one letter are not significantly different according to Tukey’s multiple comparison test (*p* < 0.05). (**b**) Main-effects plot showing the overall effect of substrate type on biomass (dry weight) of the aerial parts.

In tomato, the Pp + Bsp, Pp + Azo and Azo + Bsp consortia produced greater increases in dry weight in the commercial substrate: dry weight increased by 208.65%, 191.50%, and 274.87%, respectively, relative to the control. The growth-promoting effect was even more pronounced in greenhouse soil, where the same consortia enhanced dry weight by 531.27% (Pp + Bsp) and 406.95% (Azo + Bsp) (Figure 10a). The commercial substrate was the medium that exerted the most influence (Figure 10b).

### 2.6. Initial NPK Values in Substrates

Initial values of N (nitrogen), P (phosphorus), and K (potassium) were measured to obtain a comprehensive assessment of soil health and potential; the results are shown in Table 2.

## 3. Discussion

The results show that specific microbial consortia enhanced plant growth parameters in the three species evaluated (blue maize, broccoli, and tomato), although the degree of promotion varied depending on the bacterial combination and substrate type. In general, inoculated treatments showed trends toward increased root and stem development as well as biomass accumulation compared to uninoculated controls. However, stem elongation did not always correspond to proportional increases in biomass accumulation, suggesting that bacterial inoculation may have influenced patterns of biomass allocation and cell expansion, potentially through phytohormone-mediated growth responses. These parameters were therefore collectively interpreted to provide a complementary assessment of overall plant growth rather than as directly proportional indicators of plant performance. Notably, the magnitude of the growth-promoting response differed across specific consortium compositions and substrate conditions. This suggests that the observed growth outcomes are modulated by the growth substrate and operational conditions. Further molecular, histological, and microbiome dynamics studies are required to directly evaluate plant/microbe interactions and substrate ecological effects.

### 3.1. Bacterial Growth Kinetics and Strain Behavior

Characterizing bacterial growth kinetics is essential for the development of microbial inoculants because physiological activity, metabolite production, and cell viability are closely associated with the bacterial growth phase. Particularly, IAA production has been reported to increase during the transition from the exponential to the stationary phase of bacterial growth [19], when cellular metabolism remains highly active. Conversely, a significant kinetic parameter is the specific growth rate, which could be used to optimize and scale various industrial and environmental factors [20].

The growth curves obtained for the four bacterial strains showed that the target concentration (1 × 10^6^ cells mL^−1^) was attained while cultures were still in the exponential phase (Figure 1). This is particularly advantageous because cells in this phase generally exhibit higher metabolic activity and physiological fitness than stationary-phase cells, potentially improving their ability to colonize the rhizosphere after inoculation.

The strain used in the present study (*Mycobacterium* sp. CDBB B-77) exhibited a growth rate of 0.2530 h^−1^, indicating a rapid growth behavior under the experimental conditions evaluated. According to the CDBB culture collection (CINVESTAV-IPN, México), strain CDBB B-77 corresponds to ATCC 29472. This strain has been identified by Noh et al. [21] as *Mycobacterium fortuitum*, a rapidly growing species within the genus [22]. Thus, the growth behavior observed for *Mycobacterium* sp. CDBB B-77 is consistent with the rapid-growth characteristics reported for *M. fortuitum*; however, this observation should not be interpreted as evidence that the strain belongs to this species.

### 3.2. The Role of Phytohormone Production in Plant Growth Promotion

The bacterial strains used in this study, *Azospirillum brasilense*, *Pseudomonas putida*, *Bacillus* sp., and *Mycobacterium* sp., have been reported to possess plant-growth-promoting characteristics, including the potential to produce indole-3-acetic acid (IAA) [23,24,25,26]. IAA is involved in several aspects of plant development, including root growth and development [27]. In the present study, microbial inoculation influenced main root growth in the evaluated crops (blue maize, tomato, and broccoli) although the magnitude and statistical significance of the responses varied among consortium composition and substrate conditions (Figure 2, Figure 3 and Figure 4). These variable responses are compatible with the multiple plant-growth-promoting mechanisms reported for PGPB; however, IAA production was not quantified in the present study, and therefore its contribution to the observed root responses cannot be directly established.

Notably, several consortia containing *Azospirillum brasilense* produced favorable growth responses, although their effects varied among crop species and substrate conditions. Previous studies have reported that inoculation with *A. brasilense* can enhance plant emergence, vigor, biomass, and root-system development [28]. These reports provide a possible explanation for the responses observed in the present study, although other plant-growth-promoting mechanisms may also have contributed. In addition to IAA, bacterial production of other phytohormones, nutrient mobilization, and interactions with the plant rhizosphere may influence plant growth [25].

### 3.3. Overall Performance of Microbial Consortia

#### 3.3.1. Blue Maize

In blue maize, main root growth was strongly driven by specific microbial combinations in commercial substrate, particularly *Azospirillum brasilense* + *Bacillus* sp., *Pseudomonas putida* + *Azospirillum brasilense* and *Pseudomonas putida* + *Bacillus* sp., which increased main root growth by 61.68%, 59.61% and 40.41%, respectively (Figure 2). Although commercial substrates provided more favorable overall conditions for main root growth, consortium composition also played a meaningful role, as indicated by the significant substrate × consortium interaction. While root lengths generally decreased in greenhouse soil, plants treated with *Pseudomonas putida* + *Bacillus* sp. still outperformed the greenhouse control by 28.45% (Figure 2).

A similar context-dependent pattern was observed for stem growth. In commercial substrate *Pseudomonas putida* + *Bacillus* sp., *Azospirillum brasilense* + *Bacillus* sp., and *Mycobacterium* sp. + *Pseudomonas putida* were among the treatments producing the greatest stem responses. However, the relative performance of individual consortia varied under greenhouse soil conditions. Considering main root and stem length together, *Pseudomonas putida* + *Bacillus* sp. showed favorable responses under several experimental conditions, whereas the performance of *Azospirillum brasilense* + *Bacillus* sp. was more dependent on the evaluated variable and substrate (Figure 2 and Figure 5).

In contrast, shoot dry weight in blue maize showed a different response pattern. Several consortia containing *Mycobacterium* sp. produced relatively high dry-weight values, particularly under greenhouse soil conditions (Figure 8), consistent with some studies where *Mycobacterium* favored a total biomass accumulation [29]. These observations indicate that elongation and biomass accumulation did not necessarily respond similarly to bacterial inoculation. However, the physiological mechanisms underlying these differences were not evaluated in the present study.

#### 3.3.2. Broccoli

In broccoli, *Azospirillum brasilense* + *Bacillus* sp., *Pseudomonas putida* + *Azospirillum brasilense* and *Pseudomonas putida* + *Bacillus* sp. consortia showed the highest mean main root lengths in commercial substrate and belonged to the same Tukey group (E). Under greenhouse soil conditions, *Pseudomonas putida* + *Azospirillum brasilense* showed the highest mean main root length, whereas *Mycobacterium* sp. + *Pseudomonas putida* and *Mycobacterium* sp. + *Azospirillum brasilense* showed intermediate responses, with partially overlapping Tukey groups. As observed for blue maize main root growth, commercial substrate was associated with greater overall main root length (Figure 2). However, in broccoli, *Pseudomonas putida* + *Azospirillum brasilense* significantly increased main root length relative to the corresponding control under greenhouse soil conditions, indicating that this bacterial combination retained a growth-promoting effect under this substrate condition (Figure 3).

Regarding stem growth, the response mirrored the pattern observed in blue maize dry weight, where *Mycobacterium*-containing treatments exerted the strongest influence. In commercial substrate, the highest performance corresponded to the four-strain consortium (*Mycobacterium* sp. + *Pseudomonas putida* + *Azospirillum brasilense* + *Bacillus* sp.) and all four *Mycobacterium*-containing combinations. In this case, greenhouse soil promoted a greater overall effect on stem length, with *Mycobacterium* sp. + *Bacillus* sp. displaying the best combined response across both substrate types (Figure 6).

Finally, shoot dry weight accumulation in broccoli exhibited a dual response characterized by highly significant soil-by-consortium interaction. In commercial substrate, *Azospirillum brasilense* + *Bacillus* sp. and *Pseudomonas putida* + *Azospirillum brasilense* produced high dry-weight responses. Conversely, *Mycobacterium* sp. + *Pseudomonas putida*, *Mycobacterium* sp. + *Azospirillum brasilense*, and the four-strain consortium showed relatively greater responses in greenhouse soil. Among these treatments, *Mycobacterium* sp.+ *Pseudomonas putida* produced one of the highest shoot dry-wight values under greenhouse soil conditions (Figure 9).

#### 3.3.3. Tomato

In tomato main root development, the *Azospirillum brasilense* + *Bacillus* sp. and *Pseudomonas putida* + *Azospirillum brasilense* consortia achieved the highest growth rate in commercial substrate, while *Pseudomonas putida* + *Azospirillum brasilense* stood out in greenhouse soil. As observed in blue maize and broccoli, commercial soil yielded the greater main effect overall. Notably, *Pseudomonas putida* + *Azospirillum brasilense* showed similar mean main root length values in both substrate types, suggesting a relatively stable response under the two substrate conditions evaluated (Figure 4).

Regarding stem growth in tomato, Tukey’s multiple comparison test indicated that commercial substrate had the greatest overall impact, with *Mycobacterium* sp. + *Azospirillum brasilense* performing significantly better than the control (Figure 7).

Shoot dry weight in tomato showed a distinct response pattern compared with the other crop species, with the greatest values generally associated with consortia without *Mycobacterium* sp. In commercial substrate, *Azospirillum brasilense* + *Bacillus* sp., *Pseudomonas putida* + *Bacillus* sp., and *Pseudomonas putida* + *Azospirillum brasilense* showed the highest mean shoot dry weights and belonged to the same Tukey group (F). Under greenhouse soil conditions, *Pseudomonas putida* + *Bacillus* sp. showed the highest mean shoot dry weight, followed by *Azospirillum brasilense* + *Bacillus* sp., whereas *Pseudomonas putida* + *Azospirillum brasilense* showed a more moderate response. Overall, greater shoot dry weight was associated with commercial substrate; however, treatment-specific response differed between substrates, highlighting the influence of consortium composition on tomato biomass accumulation under the evaluated conditions. (Figure 10).

### 3.4. Functional Interactions Within Consortia

An important observation in this study is that performance varied among the evaluated treatments depending on the crop species, growth variable, and substrate. A consistent pattern emerged in consortia containing *Bacillus* sp., particularly when paired as dual combinations with *Pseudomonas putida* and *Azospirillum brasilense.* These combinations produced some of the strongest plant growth responses, consistently driving main root and stem elongation across blue maize, broccoli and tomato. *Bacillus* species, particularly *Bacillus subtilis,* are well known for their beneficial effects on both the rhizosphere microbiome and the host plant. The beneficial traits of *Bacillus* sp., particularly their capacity for efficient root colonization, stable biofilm formation, and plant growth promotion through multiple mechanisms [30], may have played a role in the positive responses documented in the present study.

However, treatment responses were also strongly shaped by soil conditions and crop species. While consortia without *Mycobacterium* sp. (such as Azo + Bsp and Pp + Azo) dominated structural elongation and dry weight in commercial substrate, a distinct functional shift occurred in dry weight accumulation. In blue maize and broccoli cultivated in greenhouse soil, *Mycobacterium*-based consortia (specifically My + Pp) achieved the highest dry-weight values. Conversely, in tomato, dry weight was favored by combinations without *Mycobacterium* sp.

The differences observed among consortium configurations may reflect interactions among the inoculated microorganisms and their responses to the plant and growing environment. Previous studies have described several mechanisms through which *Bacillus*, *Pseudomonas*, and *Azospirillum* species may influence plant growth, including root colonization, nutrient mobilization, and the production of growth-promoting compounds [31,32]. However, these mechanisms were not directly evaluated in the present study. Therefore, the observed differences among consortia cannot be attributed to a specific microbial interaction or mechanism based on the current data.

The lower responses observed for some consortia, particularly those containing *Mycobacterium* sp. (tomato dry weight), may indicate that consortium performance depends on the specific strain composition and the experimental context. However, the present study did not include direct assays of microbial interactions, such as inhibition, biofilm formation, or rhizosphere colonization. Consequently, potential antagonistic or competitive interactions among strains remain possible explanations that require direct experimental evaluation.

Furthermore, complex combinations such as the four-strain consortium did not show the greatest effect in many responses. This indicates that functional compatibility between specific strains and their adaptation to the soil context are far more critical for agricultural performance than simply increasing microbial diversity within an inoculant.

### 3.5. Influence of Substrate Type

A practical aspect of this study was evaluating bacterial inoculants under two contrasting growth media: a commercial substrate (peat moss/perlite mix) and non-sterilized greenhouse soil. Evaluating in non-sterile or commercial media provides insights closer to practical agricultural conditions.

Among the agronomic and environmental factors that affect the effectiveness of biofertilization are temperature, humidity, acidity and other chemical components of the soil, such as the content of N, P, Ca, S, Mg, Mo, Fe and Co; these factors can rapidly decrease the population of any introduced microbial species [33].

Baseline nutrient profiles varied between media (Table 2). Greenhouse soil exhibited initial concentrations of nitrogen, phosphorus, and potassium within or slightly above optimal reference ranges for plant growth (20–40 mg kg^−1^ for N, 20–40 mg kg^−1^ for P, and 150–300 mg kg^−1^ for K) [34], whereas the commercial substrate showed values close to the reference ranges of initial N and P levels. In contrast, the commercial substrate presented adequate initial N and P levels, but a lower potassium content, falling below the optimal 150 mg kg^−1^ [34]. This relative K deficit in the commercial substrate aligns with standard formulations for germination and early seedling development, where high ionic concentrations are avoided to prevent osmotic stress and cation antagonism (such as Ca^2+^ or Mg^2+^ suppression) in young, delicate root systems [35,36]. Given that this study was concluded during the initial vegetative stage, the relative K deficit in commercial substrate was not considered critical. Furthermore, evaluating the commercial substrate alongside the greenhouse soil provides insights under initial biotic states (pre-sterilized vs. non-sterilized).

However, due to baseline physicochemical differences between media, including NPK variations and contrasting physical properties—greenhouse soil is a silt-dominant soil whereas commercial substrate resembles a sandy, highly porous artificial substrate [37]—comparisons between substrates are unfeasible. Consequently, each treatment was strictly evaluated against its corresponding control to isolate true microbial effects from baseline matrix effects. From a methodological standpoint, this dual-control approach successfully fulfills the study’s primary objective: assessing microbial consortium performance and plant-growth-promoting efficacy across two distinct physical matrices and contrasting biotic states (pre-sterilized vs. non-sterilized) [38].

The differences observed between the two substrates may reflect variations in environmental conditions and native microbial dynamics. For instance, the marked growth response in commercial substrate rather than greenhouse soil (Figure 2, Figure 3, Figure 4, Figure 5, Figure 9 and Figure 10) could be linked to reduced initial competition following sterilization, which may favor early strain establishment.

In contrast, the *Pseudomonas putida* and *Bacillus* sp. consortium maintained high performance in non-sterile greenhouse soil, which may be associated with the well-documented ability of *P. putida* to colonize roots, form biofilms and persist in environments with established microbial communities [39]. It is important to mention that in the experiments carried out in greenhouse soil, there was a presence of native microbiota and greater competition for niches and resources. All these results suggest that substrate conditions can influence the effectiveness of plant-growth-promoting microorganisms.

Overall, growth trends related to controls appeared in both media, suggesting that specific microbial consortia can remain active across different substrate conditions. Nevertheless, field-scale trials and further microbiological analyses are needed to confirm how these inoculants perform under full open-field production settings.

### 3.6. Crop-Specific Responses

Although a general positive effect was observed, plant response varied among species. Tomato showed the highest increases relative to control, particularly in biomass (274.87% Azo + Bsp in commercial substrate and 531.27% Pp + Bsp in greenhouse soil) (Figure 10). In contrast, blue maize showed notable increases in main root length (61.68% for Azo + Bsp in commercial substrate; Figure 2) and shoot dry weight (147.85% for My + Pp in greenhouse soil; Figure 8). Broccoli exhibited the greatest overall effect on stem length, with increases up to 103.41% for My + Pp and 97.80% for My + Azo in greenhouse soil (Figure 6). These differences may be related to physiological traits specific to the plant, such as root architecture, exudate composition, and sensitivity to phytohormones. This variability emphasizes the importance of considering crop specificity when designing microbial inoculants [39].

This study’s findings indicate higher growth percentages in several cases when compared to previous reports. For instance, *Pseudomonas putida* has been reported to increase root and stem growth in white maize by 8% and 11% respectively when grown in sterilized soil [40]. However, in this study higher values were observed when *Pseudomonas putida* was utilized in consortia (Figure 2, Figure 3, Figure 4, Figure 5, Figure 9 and Figure 10).

Similarly, *Azospirillum brasilense* has been reported to increase white maize growth by up to 36% under sterile conditions [12], while in this work higher values were obtained under non-sterile conditions. In a previous study, the application of *Bacillus* spp., *Bacillus megaterium* and *Bacillus lichenformis* to broccoli cultivated in greenhouse soil resulted in a positive effect on biomass (dry weight) of 120% and 160%, respectively, compared with the control [41], comparable to the values observed in this study (154.06%) for the use of the *Azospirillum brasilense* and *Bacillus* sp. consortium in broccoli cultivated in commercial substrate (Figure 9).

In tomato, previous studies reported limited or no effect on biomass when using *Bacillus* and *Azospirillum* in combination [17,40], whereas in this work significant increases (*p* < 0.05) were observed in *Azospirillum brasilense* and *Bacillus* sp. in commercial substrate (274.87%) (Figure 10). These differences could be attributed to strain variability, plant genotype, or improved microbial interactions within the consortia evaluated.

### 3.7. Potential Agronomic Relevance and Study Limitations

From a practical perspective, the results of this study indicate that selected plant PGPB consortia have potential relevance for promoting early-stage plant growth under controlled laboratory conditions, although response efficacy is highly crop- and strain-dependent. Specific inoculants significantly (*p* < 0.05) promoted root length, stem extension, and biomass accumulation across the evaluated crops, with pronounced early growth effects observed in blue maize, broccoli, and tomato. Overall, the tested consortia exhibited higher growth responses in multiple plant species to varying degrees, demonstrating their potential relevance for early-stage growth promotion, pending field-level validation. These findings align with previous reports on the use of PGPB, which frequently display broad-spectrum effectiveness across diverse host plants [39]. It is worth noting that performance trends varied depending on the complexity of the inoculant and the growth substrate.

The four-strain consortium did not consistently outperform simpler combinations, and in some cases, it demonstrated reduced effectiveness. This suggests that the interaction between the strains is not purely additive and that simpler consortia may outperform more complex mixtures.

## 4. Materials and Methods

### 4.1. Experimental Site and Biological Material

The research was conducted at the Environmental Technology Laboratory of the Unidad Profesional Interdisciplinaria de Biotecnología, Instituto Politécnico Nacional. The plants used in the study were cultivated in a greenhouse.

The microorganisms used in this study (Table 3) were obtained from the National Collection of Microbial Strains and Cell Cultures (known as CDBB) at the Center of Research and Advanced Studies of the National Polytechnic Institute (CINVESTAV-IPN) in G.A.M., Mexico City, Mexico. The seeds used were tomato (*Solanum lycopersicum*) and broccoli (*Brassica oleracea*) seeds acquired from Hydro Environment. Blue maize (*Zea mays*) seeds were obtained directly from local farmers in Estacion de Apulco, Hidalgo, Mexico.

The four strains were selected based on their availability within the CDBB collection and the previously reported plant-growth-promoting potential of their respective genera.

The commercial substrate was 1:1 perlite–peat moss mixture (Hydro Environment company, Tlalnepantla, State of Mexico, Mexico) and greenhouse soil was provided from tomato farmers in Hidalgo, Mexico. To ensure a homogeneous nutrient supply, a commercial hydroponic nutrient solution was used (Intergarden^®^, Guadalajara, Jalisco, Mexico) during the inoculation.

### 4.2. Formulation of Culture Media

Three culture media were used for bacterial cultivation. *Bacillus* sp. and *Pseudomonas putida* were grown in Nutrient Broth (BD Bioxon^®^, Cuatitlan Izcalli, Satate of Mexico, Mexico) prepared at a concentration of 8 g L^−1^ in distilled water and adjusted to pH 6.8. *Azospirillum brasilense* was grown in Mannitol Broth adjusted to pH 6.0, whereas *Mycobacterium* sp. was grown in Emerson Broth, adjusted to pH 6.2. The compositions of the Mannitol and Emerson media are presented in Table 4 and Table 5, respectively.

### 4.3. Formulation of Microbial Consortia

Microbial growth kinetics were evaluated in a batch culture system using 250 mL Erlenmeyer flasks with a working volume of 100 mL. Growth kinetics were monitored starting from an initial density of 1 × 10^4^ cells mL^−1^ up to 1 × 10^6^ cells mL^−1^. This range allowed us to capture the active exponential growth phase, ensuring optimal metabolic activity and cell viability at harvest while directly matching the targeted working density for plant inoculation. Cultures were incubated at 35 °C under constant orbital agitation at 120 rpm. Bacterial growth was measured by direct cell counting using a Neubauer chamber. The growth assay was performed in triplicate (n = 3).

The specific growth rate (h^−1^) was calculated based on the exponential equations, calculated using data points from the exponential growth phase of each bacterial kinetic.

An inoculation titer of 1 × 10^6^ cells mL^−1^ per strain was selected based on evidence showing that moderate PGPR inoculation levels effectively promote plant responses and rhizosphere colonization without inducing unnecessary perturbations in native soil microbial functional groups [42]. Biomass was harvested by centrifugation at 6000 rpm for 10 min; the resulting cell pellet was resuspended in a sterile nutrient solution. The final bacterial concentration was 2 × 10^6^ cells mL^−1^.

### 4.4. Evaluation of Agronomic Parameters

Broccoli and tomato seeds were germinated in trays and maize in polyethylene bags using the corresponding soil. The experiment was conducted under greenhouse conditions (25–30 °C) with a natural daylight photoperiod for 26 days, 6 days until germination and 20 days after the inoculation.

A total of eight treatments were evaluated, including seven microbial consortia and one control (Table 6). Each treatment consisted of three replicates, with five plants per replicate. A single application of each microbial consortium (Table 6) was performed independently for each plant species. Six days after germination, the bacterial suspension was applied directly to the soil at a volume of 5 mL per plant for tomato and broccoli, and 15 mL per plant for blue maize. The consortium concentration was 1 × 10^6^ cells mL^−1^ for each bacterial strain. Control plants received an equivalent volume of sterile nutrient solution.

Twenty days after the inoculation, plants were harvested to assess dry weight, main root length, and longitudinal growth. These parameters were measured directly with a millimeter ruler, and dry weight was determined using a thermobalance until constant weight was achieved.

All the consortium biomasses were resuspended in sterile nutrient solution (Intergarden^®^). As for the control, we used sterile nutrient solution (Intergarden^®^) without cell inoculum.

### 4.5. Determination of Substrate Macronutrients (NPK)

To extract the soluble fractions of nitrogen, phosphorus and potassium, dry, sieved soil was suspended in distilled water (1:20 *w*/*v* ratio) [43]. The mixture was allowed to stand for one hour and was subsequently filtered to eliminate turbidity and suspend solids [44].

Phosphorus and potassium quantifications were performed digitally using a Hanna Instruments^®^ (Woonsocket, RI, USA) HI 83099 multiparameter photometer. For potassium, the commercial HI93750-01 kit, based on the tetraphenylborate turbidimetric method, was utilized. For phosphorus, the HI93763B-50 high-range digestion kit was employed following the vanadomolybdophosphoric acid method [45].

Conversely, the nitrogen measurement was analytically independent. Samples were processed using HI93767B-50 persulfate digestion vials (chromotropic acid method). Absorbance readings were executed on a spectrophotometer, and final concentrations were determined via linear interpolation against a calibration curve previously standardized in the laboratory [45]. The pH was also measured in a 1:2 soil–water suspension using a calibrated pH meter. All tests were performed in triplicate.

### 4.6. Experimental Design and Statistical Analysis

The treatments were arranged in a randomized design for the formation of bacterial pair consortia to ensure the independence of experimental errors. The data obtained were evaluated using a General Linear Model (GLM) to analyze main effects and interactions across substrates. Post hoc comparisons were conducted using Tukey’s Honest Significant Difference (HSD) test at a confidence level of 95% (α = 0.05), performed across both substrates.

All statistical analyses, post hoc evaluations, and creation of interaction graphics were conducted using Python (version 3.14.7, Python Software Foundation, Wilmington, DE, USA). Data processing and structured summary outputs were managed with pandas (v3.0.5) and NumPy (v2.5.2). To assess treatment mean separations, pairwise comparisons were performed via Tukey’s (HSD) test implemented using the statsmodels.stats.multicomp.MultiComparison module (Statsmodels, v0.14+).

Compact Letter Display (CLD) assignments for post hoc groupings were constructed using maximal clique analysis provided by the NetworkX library (v3.6.1).

Kinetic bacterial plots were made with SciDAVis (version 2.0+, free open-source software).

Bar charts plots were generated using Origin (version 10.3, OriginLab Corporation, Northampton, MA, USA).

## 5. Conclusions

Once all factors were considered (main root growth, stem longitudinal growth, and dry weight), it was determined that the consortia with the best values contained *Pseudomonas putida* (*Pseudomonas putida* with *Bacillus* sp., *Pseudomonas putida* with *Azospirillum brasilense* and *Mycobacterium* sp. with *Pseudomonas putida*).

In contrast, the four-strain combination did not yield proportional enhancements in plant growth, indicating that increasing microbial diversity to four strains did not necessarily result in greater effectiveness and sometimes diminished overall treatment effectiveness with the strains evaluated in this study.

The enhanced performance observed in two specific strain combinations may be attributed to complementary mechanisms such as phytohormone production, nutrient solubilization, and induction of systemic resistance. Notably, increasing the consortium complexity to four strains did not consistently yield the highest impact across all evaluated parameters, although the present experimental design does not allow for the determination of the specific contribution of individual strains or the presence of synergistic or antagonistic interactions. In addition, these findings indicate that the design of effective microbial consortia should be based on functional diversity, rather than simply increasing the number of strains.

Evaluations across substrate conditions influenced both plant growth and the relative efficacy of the microbial inoculants. Initial NPK characterization suggested sufficient baseline nutrient levels in both media, ensuring that nutritional deficiencies did not interfere with plant growth responses. Overall, higher growth enhancement was observed in the sterile commercial substrate, whereas specific combinations (such as consortia containing *Mycobacterium* sp.) yielded prominent responses in the heterogeneous greenhouse soil. These performance variations between media could be attributed to the contrasting nature of a pre-sterilized commercial substrate versus an unsterilized one.

The results obtained with consortia containing *Mycobacterium* sp. provide preliminary insights into crop growth promotion under the evaluated conditions. However, lacking species-level taxonomic confirmation and given the potential association with opportunistic species such as *Mycobacterium fortuitum*, its field application or inclusion in biostimulant formulations is not recommended until rigorous biosafety assessments are completed. Additional taxonomic confirmation of *Bacillus* sp. would enhance the characterization of the isolates used.

The findings indicate that the success of PGPB-based strategies relies on selecting effective strain combinations and validation under realistic soil conditions. These results support the potential of tailored microbial consortia in early seedling vigor in sustainable agriculture. Future studies should prioritize field validation, mechanistic analysis of plant–microbe interactions, and optimization of formulations for large-scale agricultural use.

## Figures and Tables

**Table 1:** Growth kinetic parameters of the evaluated bacterial strains.

Microorganism	Specific Growth Rate (h^−1^)	Time to Reach 1 × 10^6^ Cells mL^−1^ (h)
*Azospirillum brasilense*	0.3462	7
*Bacillus* sp.	0.3184	8
*Pseudomonas putida*	0.3496	7
*Mycobacterium* sp.	0.2530	14

**Table 2:** Initial values of NPK in commercial substrate and greenhouse soil.

Parameters	Commercial Substrate (ppm)	Greenhouse Soil (ppm)
N (nitrogen)	25.23 ± 1.7	45.17 ± 1.97
P (phosphorus)	22.67 ± 3.06	32 ± 3.46
K (potassium)	79.33 ± 1.15	310
pH	6	6

**Table 3:** Bacterial strains used in this study, including their CDBB collection numbers and corresponding ATCC reference strains.

Microorganism	Strain	Acronym
*Azospirillum brasilense*	CDBB B-1887	ATCC 29145
*Bacillus* sp.	CDBB B-551	ATCC 9053
*Pseudomonas putida*	CDBB B-1299	ATCC 12633
*Mycobacterium* sp.	CDBB B-77	ATCC 29472

**Table 4:** Composition of Mannitol Broth.

Component	Concentration (g L^−1^)	Brand
Peptone	3	Thermo Fisher^®^ (Waltham, MA, USA)
Yeast extract	5	BD Bioxon^®^
Mannitol	25	Sigma Aldrich^®^ (St. Louis, MO, USA)

**Table 5:** Composition of Emerson Broth.

Component	Concentration (g L^−1^)	Brand
Yeast extract	1	BD Bioxon^®^
NaCl	2.5	Anasol^®^ (Iztacalco, Mexico City, Mexico)
Peptone	4	Thermo Fisher^®^
Meat extract	4	BD Bioxon^®^
Dextrose	20	Alyt^®^ (V. Carranza, Mexico City, Mexico)

**Table 6:** Bacterial consortia evaluated in the growth of tomato, broccoli, and corn plants.

Microbial Consortium	Strain
Control	C
*P. putida* + *Bacillus* sp.	Pp + Bsp
*P. putida* + *A. brasilense*	Pp + Azo
*A. brasilense* + *Bacillus* sp.	Azo + Bsp
*Mycobacterium* sp. + *A. brasilense*	My + Azo
*Mycobacterium* sp. + *P. putida*	My + Pp
*Mycobacterium* sp. + *Bacillus* sp.	My + Bsp
*Mycobacterium* sp. + *P. putida* + *A. brasilense* + *Bacillus* sp.	My + Pp + Azo + Bsp

## Data Availability

The original contributions presented in this study are included in the article. Further inquiries can be directed to the corresponding authors.
